# Dementia Caregiving: The Association Between Family Caregiving Strain and Receipt of Paid Care

**DOI:** 10.1093/geroni/igaa057.1898

**Published:** 2020-12-16

**Authors:** Jennifer Reckrey, Evan Bollens-Lund, Emma Tsui, Kathrin Boerner, Katherine Ornstein

**Affiliations:** 1 Icahn School of Medicine at Mount Sinai, New York, New York, United States; 2 CUNY Graduate School of Public Health & Health Policy, New York, New York, United States; 3 University of Massachusetts Boston, Boston, Massachusetts, United States

## Abstract

family caregivers. Using data from three waves (2011, 2015, 2017) of the National Health and Aging Trends Study (NHATS) linked to the National Study of Caregiving (NSOC), we identified family caregivers of those with advanced dementia and compared caregiving strain among those with zero, <20, and 20+ hours/week paid care. Family caregivers of those who received 20+ hours (26% of the sample) reported less caregiver strain (mean score 3.27 vs 4.15, p=0.04) and less frequently reported having more to do than they could handle (46.1% vs 67.9%, p=0.01) or not having enough time for themselves (46.8% vs 72.2%, p<0.01). The association persisted in a multivariable model. These results support the conceptualization of dynamic and potentially multidirectional relationships between paid and family caregivers and suggest that paid caregivers impact both those receiving care and their families.

